# Adapting MR-BrainPET scans for comparison with conventional PET: experiences with dynamic FET-PET in brain tumours

**DOI:** 10.1186/2197-7364-1-S1-A64

**Published:** 2014-07-29

**Authors:** Philipp Lohmann, Hans Herzog, Elena Rota Kops, Gabriele Stoffels, Christian Filss, Norbert Galldiks, Heinrich H Coenen, N Jon Shah, Karl-Josef Langen

**Affiliations:** Institute of Neuroscience and Medicine (INM-3,-4,-5), Forschungszentrum Juelich, Juelich, Germany; Department of Neurology, University of Cologne, Cologne, Germany

Imaging results from subsequent measurements (preclinical 3T MR-BrainPET, HR+) are compared. O-(2-[^18^F]fluoroethyl)-L-tyrosine (FET) may exhibit non-uniform tracer uptake in gliomas. The aim was to analyse and adapt the physical properties of the scanners and study variations of biological tumour volume (BTV) in early and late FET-PET.

Spatial resolution of the BrainPET and HR+ was measured according to NEMA standard. For evaluation of a threshold-based volume determination -as used for BTV- volumes of an ^18^F-filled spheres phantom were evaluated. Influence of different filter kernels for correction of differences in spatial resolution hereon was compared.

Differences in BTV between early and late FET-PET of 45 patients were analysed. BTV was determined using a tumour-to-brain ratio ≥1.6 [1].

Spatial resolution (FWHM) of the BrainPET was 2.63mm–3.47mm and 4.39mm–5.10mm for the HR+ (10mm off-centre) [2]. 3D-filtered backprojection was used for reconstruction [3]. BTV of largest sphere was 22.8ml in HR+ and between 23.2ml (unfiltered) and 24.5ml (3D-Gaussian 3.5mm) in the BrainPET. BTV of smallest sphere was 0.1 ml in HR+ and between 0.2ml (unfiltered) and 0.06ml (3D-Gaussian 3.5mm) in the BrainPET. A 2.5mm filter showed the smallest deviation for all spheres and was applied to the BrainPET data for cross-scanner comparison. Changes in BTV >10% were considered significant and not related to physical differences between scanners.

41% of patients showed a considerable deviation between early and late FET-PET. BTV increased in 14 patients. Four patients showed a FET positive region only in late FET-PET.

Taking into account the physical differences of PET scanners is important for cross-scanner studies. It was shown in a patient study that BTV may vary between early and late FET-PET, which is important for patient management and needs further investigation.

**Figure 1:**
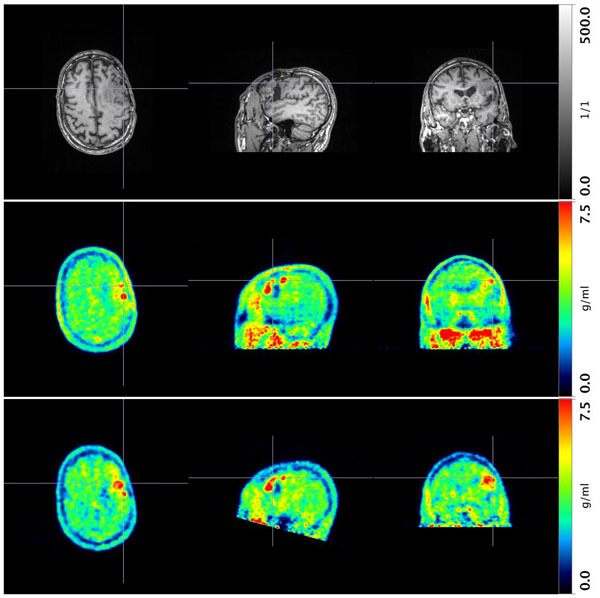
MRI and FET-PET scan of a patient with oligodendroglioma WHO grade II. T1-weighted MR image (MPRAGE) acquired simultaneously to the early PET scan with the 3T MR-BrainPET (top). Summation scan 20-40 min post-injection (BrainPET, middle), summation scan 70-90 min post-injection (HR+, bottom). Volume of FET-positive region (frontal) increased from 3.8ml to 10.7ml.
